# Supplementary material for: Normo- or Hypo-Fractionated Photon or Proton Radiotherapy in the Management of Locally Advanced Unresectable Pancreatic Cancer: A Systematic Review
Source: Cancers (Basel). 2023 Jul 25;15(15):3771. doi: 10.3390/cancers15153771 (PMC10416887; doi:10.3390/cancers15153771)
Supplement: Supplementary file 1 [file cancers-15-03771-s001.zip › cancers-2468971-supplementary.pdf]

cancers

MDPI

Systematic Review

Normo- or Hypo-Fractionated Photon or Proton Radiotherapy in the Management of Locally Advanced Unresectable Pancreatic Cancer: A Systematic Review

Supplementary Materials

Bias Domain's evaluation: Each study was rated as low, high, or unclear risk according to the following domains: major domains (biases 1–4) and minor domains (biases 5 and 6). A study was overall ranked as low risk of bias, meaning high quality, if all the major domains were scored as low risk. If any of the major domains were rated as high or unclear risk, then the study was classified as having an overall high risk.

Bias in selection of participants and recruitment:

Low risk: Patients' assignment or randomization to either proton or photon therapy was not affected by financial ability, patient decision, or availability of the treatment technique.

High risk: Patient selection for a specific therapy was affected by the financial ability, patient decision, or availability of the technique.

Bias in clarification of interventions:

Low risk: Details on radiation therapy (fractionation schedule, technique) were clearly defined, chemotherapy protocols were clearly reported, or follow-up scheduling (timing and investigations) are clearly reported and appropriate.

High risk: Wide range of unspecified radiation fractionation schedules, wide range of unspecified radiation techniques, Use of different, unspecified chemotherapy protocols or no follow-up scheduling (timing and investigations) reported.

Bias in measurement of outcomes:

Low risk: The toxicity profile assessment tool was clearly defined or adequate descriptive statistics, e.g.: acute toxicity, chronic toxicity and median overall survival were reported with range or confidence interval for the patient group.

High risk: The toxicity profile assessment tool was not clearly defined or inadequate statistical reporting.

Bias due to missing of the reported data:

Low risk: Main outcome parameter (median overall survival), patient and tumor characteristics, e.g., age, stage and toxicity profile were reported per patient cohorts/ treatments.

High risk: Main outcome parameter (median overall survival), patient characteristics and toxicity profile were mixed for different patient cohorts / treatments.

Funding:

The studies were evaluated to be low risk if the funding is reported to be by non-profit organizations.

Conflict of Interest:

If the study authors reported not having a conflict of interest, this domain was considered low risk.
